# Supplementary material for: Adaptation of a clinical reasoning model for use in inflammatory conditions of the lactating breast: a retrospective mixed-methods study
Source: PeerJ. 2022 Jul 25;10:e13627. doi: 10.7717/peerj.13627 (PMC9332403; doi:10.7717/peerj.13627)
Supplement: Supplemental Information 1 [file peerj-10-13627-s001.docx]

| **Categories and subcategories** | **Factors** |
| --- | --- |
| **CNS modulation** |  |
| Prolonged afferent input | Prolonged nipple or breast pain |
| Predisposing factors | Parity (+/- self-efficacy) |
|  | Pain history *(including parental influence)* |
|  | Mastalgia |
|  | Previous trauma *(including sexual and physical violence)* |
|  | Pain education (confidence) |
| Cognitive – emotive – social state | Anxieties/expectations: previous bad breastfeeding experience or other negative feelings about self or baby |
|  | Personal control *(including interaction with partner)* |
|  | Social support |
|  | Fatigue and nutrition |
|  | Attention on breastfeed as new skill |
| **External influences** |  |
| Attributes of mother | Nipple shape |
|  | Flexibility of nipple |
| Attributes of infant | Tongue-tie |
|  | Small mouth |
| Interaction between mother and infant | Shallow latch |
| Miscellaneous | Breast pump |
|  | Cream |
|  | Pad |
|  | Temperature |
| **Local stimulation** |  |
| Chemical stimulation | Inflammatory mediators |
|  | Prostaglandins |
|  | Oxytocin |
|  | Catecholamines |
| Skin breakdown | (Skin breakdown) |
