# Supplementary material for: Adaptation of a clinical reasoning model for use in inflammatory conditions of the lactating breast: a retrospective mixed-methods study
Source: PeerJ. 2022 Jul 25;10:e13627. doi: 10.7717/peerj.13627 (PMC9332403; doi:10.7717/peerj.13627)
Supplement: Supplemental Information 2 [file peerj-10-13627-s002.docx]

| **Factors** | **Phase of, and reason for, exclusion** | | | |
| --- | --- | --- | --- | --- |
|  | *Phase 3: Clinical audit factors* | | *Phase 5: Proposed adapted model* | |
|  | Prior research factors, not identified within clinical audit population  *(Frequency, n = 0)* | Existing model factors, not identified within clinical audit population  *(Frequency, n = 0)* | | Iterative process criteria  *(No underlying physiological significance/ rationale/ mechanism)* |
| **CNS Modulation** | | | | |
| **Cognitive – emotive – social state** | | | | |
| Attention on breastfeed as a new skill |  | X | |  |
| History of sexual abuse | X |  | |  |
| Maternal health: |  |  | |  |
| Psychological mood |  |  | | X |
| Throat infection | X |  | |  |
| Personal control |  | X | |  |
| Social support |  | X | |  |
| **Pre-existing** |  | | | |
| Age (maternal) |  |  | | X |
| Childbirth location |  |  | | X |
| Education | X |  | |  |
| Income | X |  | |  |
| Mastalgia |  | X | |  |
| Multiple birth | X |  | |  |
| Pain education |  | X | |  |
| Pain history |  | X | |  |
| Smoking | X |  | |  |
| **External influences** | | | | |
| **Attributes of infant** | | | | |
| Birth weight ≤ 3500 gms |  |  | | X |
| Infant age |  |  | | X |
| **Interaction between mother and infant** | | | | |
| First contact with child |  |  | | X |
| Separation of mother-infant |  |  | | X |
| **Physiological attributes of mother** | | | | |
| Anemia | X |  | |  |
| Flexibility of nipple |  | X | |  |
| Menses returned | X |  | |  |
| **Miscellaneous** |  | | | |
| Clean nipple before breastfeeding | X |  | |  |
| H_2_0 1^st^ month | X |  | |  |
| Pacifiers | X |  | |  |
| Prelacteal feeding | X |  | |  |
| Supplements |  |  | | X |
| Temperature |  | X | |  |
| **Local influences** | | | | |
| **Breakdown** | | | | |
| Staphylococcus aureus | X |  | |  |
| **Stimulation** | | | | |
| Catecholamines |  | X | |  |
| Inflammatory mediators |  | X | |  |
| Oxytocin |  | X | |  |
| Prostaglandins |  | X | |  |
|  |  | | | |
