# Supplementary material for: Adaptation of a clinical reasoning model for use in inflammatory conditions of the lactating breast: a retrospective mixed-methods study
Source: PeerJ. 2022 Jul 25;10:e13627. doi: 10.7717/peerj.13627 (PMC9332403; doi:10.7717/peerj.13627)
Supplement: Supplemental Information 3 [file peerj-10-13627-s003.docx]

| **Factors** | **Identification** |
| --- | --- |
| **CNS Modulation** | |
| **Afferent input** | |
| Breastfeeding pain | BF pain (Wilson et al. 2020)  Breast pain (Amir et al. 2015) |
| Nipple injury | Nipple damage (Wilson et al. 2020)  Cracked nipple (Yin et al. 2020) (Deng et al. 2020)  Skin breakdown (Amir et al. 2015) |
| Nipple pain | Nipple pain (Wilson et al. 2020)  Nipple pain (Amir et al. 2015) |
| **Cognitive – emotive – social state** | |
| Maternal health: |  |
| Anxiety/depression | Anxiety, depression (clinical audit)  Anxieties (Amir et al. 2015) |
| Fatigue | Run down (clinical audit)  Fatigue (Amir et al. 2015) |
| Increased psychological stress | Stress level (Wilson et al. 2020) |
| Infection | Infection – Women with throat infection (Wilson et al. 2020)  Throat infection (Yin et al. 2020)  Any viral/bacterial infection (clinical audit) |
| Previous bad breastfeeding experience | Previous bad breastfeeding experience (clinical audit) (Amir et al. 2015) |
| Traumatic birth | Traumatic birth (clinical audit)  Previous trauma (Amir et al. 2015) |
| **Pre-existing** |  |
| Birth interventions | Mode of delivery (Wilson et al. 2020)  Delivery mode – caesarean section (Yin et al. 2020)  Caesarean section (Deng et al. 2020)  Induction (clinical audit)  Preterm birth (clinical audit) |
| Employed | Occupation (Wilson et al. 2020) |
| High socioeconomic status | SES (Wilson et al. 2020)  Health service access – Private health insurance (Wilson et al. 2020) |
| Multiparous | Parity (Wilson et al. 2020)  Delivery number - multiparous (Yin et al. 2020)  Parity (Amir et al. 2015) |
| PHx ICLB | Hx mastitis – in previous lactations/BF (Wilson et al. 2020)  Mastitis in previous breastfeeding (Yin et al. 2020)  Mastitis during previous breastfeeding (Deng et al. 2020)  PHx of the suite of ICLB in current and previous lactation periods (clinical audit) |
| **External influences** | |
| **Attributes of infant** | |
| Ill health | Baby – Women whose baby had a runny nose (Wilson et al. 2020)  Oral dysplasia – Oral thrush (Yin et al. 2020)  Other conditions of inferior infant health (clinical audit) |
| Oral anomaly | Oral dysplasia – Tongue tie (Yin et al. 2020)  Other oral anomalies (clinical audit)  Tongue-tie, small mouth (Amir et al. 2015) |
| **Interaction between mother and infant** | |
| Breastfeeding behaviour & practices | Consecutive same breast (Wilson et al. 2020)  BF position (Wilson et al. 2020)  BF frequency and timing: > 30 min, feeding more frequently in 48 hr before mastitis onset, feeding < 6 times/day, feeding 6-9 times/day (Wilson et al. 2020)  Breastfeeding duration > 30 min (Deng et al. 2020)  Sleeping with sucking (Yin et al. 2020)  Variable behaviour/change in regime (clinical audit) |
| Decreased milk transfer | BF frequency and timing – Decreased milk, tried to wean (Wilson et al. 2020) |
| Fit and hold (attachment & positioning) difficulty | Attachment difficulties (Wilson et al. 2020)  BF difficulty (Wilson et al. 2020)  Connection difficulty (Yin et al. 2020)  Sucking manners – Nipple and areola sucking (Yin et al. 2020)  Latch problem (Deng et al. 2020)  Shallow latch (Amir et al. 2015) |
| **Physiological attributes of mother** | |
| High milk supply | Milk supply (Wilson et al. 2020)  Over-producing milk (Deng et al. 2020) |
| Low milk supply | Low milk supply (clinical audit) |
| Milk came in >24 hrs | Milk came in > 24 hr (Wilson et al. 2020) |
| Nipple anatomy | Nipple’s heteroplasia – Nipple retraction, nipple applanation, large nipple (Yin et al. 2020)  Nipple shape (Amir et al. 2015) |
| PHx non-lactational breast complications | Breast surgery (Deng et al. 2020)  Other non-lactational breast complications (clinical audit) |
| **Miscellaneous** |  |
| Antibiotic use | Antibiotics (Wilson et al. 2020)  Oral antibiotics (Yin et al. 2020)  Peripartum antibiotic therapy (Deng et al. 2020) |
| Any pump use | Expressing milk (Wilson et al. 2020)  Breast pump (Yin et al. 2020)  Use of a breast pump (Deng et al. 2020)  Breast pump (Amir et al. 2015) |
| External nipple contacts | Use of nipple cream – Hydrogel dressings (Wilson et al. 2020)  Nipple shield (Wilson et al. 2020)  Sucking manners – Nipple shields (Yin et al. 2020)  Use of nipple shields (Deng et al. 2020)  Other nipple dressings/patches (clinical audit)  Pad (Amir et al. 2015) |
| Nipple creams | Use of nipple cream – antifungal cream (Wilson et al. 2020)  Use of cream on nipples (Deng et al. 2020)  Cream (Amir et al. 2015) |
| **Local influences** | |
| **Breakdown** | |
| Blocked ducts | Blocked ducts (Wilson et al. 2020)  Blocked duct (Deng et al. 2020) |
| Engorgement | Engorgement (Wilson et al. 2020) |
| Milk bleb | Milk bleb (clinical audit) |
| Nipple thrush | Nipple thrush (clinical audit) |
| **Stimulation** | |
| External compression | Bra wearing – tight bra (Wilson et al. 2020)  Breast trauma by external force (Yin et al. 2020)  Sleeping posture (prone and lateral position) (Yin et al. 2020)  Breast trauma (Deng et al. 2020)  Other types of external compression (clinical audit) |
| Firm massage/handling | Non-medical staff massage (Yin et al. 2020)  Firm massage/handling (clinical audit) |
